# Caracterización y factores asociados a la mortalidad debida a enfermedades huérfanas en Chile, 2002-2017

**DOI:** 10.7705/biomedica.6281

**Published:** 2022-09-02

**Authors:** Jahir Andrés Avila, Julio César Martínez

**Affiliations:** 1 Hospital Base de Valdivia, Unidad de Diálisis, Valdivia los Ríos, Chile Hospital Base de Valdivia Unidad de Diálisis Valdivia los Ríos, Chile; 2 Departamento de Salud Pública, Universidad Industrial de Santander, Bucaramanga, Colombia Universidad Industrial de Santander Departamento de Salud Pública Universidad Industrial de Santander Bucaramanga, Colombia

**Keywords:** enfermedades raras, costo de enfermedad, registros de mortalidad, salud pública, Chile, Rare diseases, cost of illness, mortality registries, public health, Chile

## Abstract

**Introducción.:**

Las enfermedades huérfanas se caracterizan por su baja prevalencia, comúnmente son de evolución crónica, debilitantes y potencialmente mortales.

**Objetivo.:**

Determinar las características y los factores asociados a la mortalidad por enfermedades huérfanas en Chile, entre 2002 y 2017.

**Materiales y métodos.:**

Es un estudio transversal y analítico a partir de datos secundarios oficiales del Departamento de Estadística e Información en Salud (DEIS) del Ministerio de Salud de Chile. Se calcularon las tasas de mortalidad específica, y las ajustadas por sexo y edad. Se efectuó un análisis de normalidad mediante la prueba de Kolmogórov-Smirnov. Se aplicaron la prueba de ji al cuadrado de independencia para las asociaciones y el análisis de regresión logística multivariada para determinar la probabilidad de muerte.

**Resultados.:**

Durante el periodo de estudio, 10.718 defunciones se atribuyeron a enfermedades huérfanas; 53,2 % ocurrieron en mujeres. La tasa media anual de mortalidad fue de 3,9 por 100.000 habitantes: 4,1 en mujeres y 3,8 en hombres. Las principales causas de muerte, en mujeres, fueron enfermedad de Creutzfeldt-Jakob, anencefalia, hepatitis autoinmunitaria y, en hombres, enfermedad de Creutzfeldt-Jakob, distrofia muscular y anencefalia. Las mujeres tienen 1,75 más veces la posibilidad de fallecer por este grupo de enfermedades en comparación con los hombres (OR ajustado=1,75; IC_95%_ 1,69-1,82). La mayor probabilidad de morir se presentó en los menores de 0 a 4 años (OR ajustado=15,30; IC_95%_ 14,10-19,20).

**Conclusión.:**

En Chile, las mujeres constituyeron el grupo de población de mayor riesgo de morir por enfermedades huérfanas durante los años 2002 y 2017.

## Abstract (en)

**Introduction:**

Rare diseases are characterized by their low prevalence, chronically debilitating and life-threatening nature.

**Objective:**

To determine the characteristics and factors associated with mortality due to rare diseases in Chile from 2002 to 2017.

**Materials and methods:**

We conducted an analytical cross-sectional study based on secondary mortality database from the *Departamento de Estadística e Información en Salud (DEIS), Ministerio de Salud de Chile* (Department of Statistics and Health Information, Chile Ministry of Health) from 2002 to 2017. The specific mortality rates adjusted by age and sex were calculated. A normality analysis was conducted using the Kolmogorov-Smirnov test. In addition, a chi-square test of independence for associations and multivariate logistic regression was applied to determine the probability of death.

**Results:**

Between 2008 and 2012 there were 10,718 deaths due to rare diseases, 53.2% of them occurred among women. The average annual mortality rate was 3.9 per 100,000 inhabitants: 4.1 in women and 3.8 in men. The main causes of mortality among women were Creutzfeldt-Jakob disease, anencephaly and autoinmune hepatitis, and among men, Creutzfeldt-Jakob disease, muscular dystrophy and anencephaly. Women are 1.75 times more likely to die than men (adjusted Odds Ratio (_a_OR) = 1.75; 95% CI: 1.69 - 1.82). The highest probability of dying occurred among children aged 0-4 years (_a_OR = 15.30; 95% CI: 14.10 - 19.20).

**Conclusion:**

Overall, the burden of mortality due to rare disease was higher among women of all ages in Chile between 2002 and 2017.

Las enfermedades huérfanas, también denominadas raras o poco frecuentes, constituyen un grupo amplio y variado de trastornos de salud que se caracterizan por su baja prevalencia y las altas tasas de mortalidad prematura ^1^. Se estima que existen de 5.000 a 8.000 enfermedades huérfanas que podrían afectar entre el 6 y el 8 % de la población mundial. El 80 % de ellas son de origen genético, del 20 al 25 % están relacionadas con enfermedades del sistema inmunológico y, en menor proporción, con neoplasias y etiologías infecciosas ^1^. Suelen ser crónicamente debilitantes y muy graves, y comprometen más de un órgano vital con múltiples deficiencias motoras, sensoriales y cognitivas.

La expectativa de vida de las personas afectadas varía ampliamente según la gravedad de la enfermedad, pero, en términos generales, es muy reducida. Un porcentaje importante de estos pacientes mueren al nacer y otro grupo de ellos desarrollan enfermedades degenerativas en el trascurso de su ciclo vital ^(^^2^. La prevalencia es mayor en adultos debido al exceso de mortalidad reportado para algunas enfermedades huérfanas en la población pediátrica, principalmente aquellas atribuidas a malformaciones genéticas ^3^.

La definición de las enfermedades huérfanas varía entre países, dependiendo del umbral en el cual una enfermedad se considere poco frecuente. Por ejemplo, la Comunidad Europea establece como enfermedad huérfana toda condición de salud cuya prevalencia sea menor de 5 casos por cada 10.000 habitantes. En Japón, una enfermedad se considera rara cuando existen menos de 50.000 (1 en 2.500) casos, mientras que, en Taiwán, cuando la prevalencia es inferior a un caso por 100.000 personas. En Estados Unidos, se utiliza una cifra menor de 200.000 casos en todo el país para clasificar un trastorno dentro de las enfermedades huérfanas ^3^. En Colombia, se define como aquellas enfermedades que presentan una prevalencia menor de un caso por cada 5.000 personas ^4^.

Chile no cuenta con una definición propia. Como antecedente normativo, se tiene el proyecto de ley sobre enfermedades poco frecuentes, en el cual el país adopta la clasificación de la Unión Europea y la de Estados Unidos. En ese marco, establece como enfermedades huérfanas a ciertas condiciones que afectan a los seres humanos y que presentan muy baja prevalencia y gran diversidad, en consonancia con las políticas internacionales que promuevan el desarrollo de fármacos para tratarlas ^5^. En ese mismo horizonte, la Federación Chilena de Enfermedades Raras, FECHER, integrada por pacientes, familiares, profesionales de la salud y personas sensibilizadas por el tema, ha participado activamente en la gestión de la Ley 20.850 de 2015, conocida como “Ley Ricarte Soto”, mediante la cual se reglamenta la atención, el diagnóstico, la cobertura universal y el tratamiento de 14 afecciones específicas de salud asociadas con enfermedades huérfanas ^6^.

Se desconoce el impacto real de la carga de morbilidad, mortalidad, tecnologías diagnósticas y terapéuticas, y las complicaciones relacionadas con la discapacidad de muchas de estas enfermedades. Esto puede deberse en gran medida a su baja prevalencia y, por consiguiente, constituyen eventos de salud nuevos para la comunidad científica de diferentes regiones y contextos. Aunado a lo anterior, se añaden dificultades con la inespecificidad de códigos usados en el sistema vigente de clasificación de enfermedades, puesto que muchas de ellas comparten la misma nomenclatura con otras enfermedades.

En ese sentido, los análisis de mortalidad representan una herramienta valiosa. Por un lado, permiten actualizar el conocimiento que se tiene sobre estas enfermedades y, por otro, brindan información pertinente para evaluar de forma indirecta los avances en relación con detección oportuna, atención, manejo y seguimiento de las condiciones de salud asociadas con enfermedades huérfanas. Por lo anterior, el objetivo de este trabajo es determinar las características y los factores asociados a la mortalidad por enfermedades huérfanas en Chile, entre 2002 y 2017.

## Materiales y métodos

Se realizó un estudio transversal y analítico mediante el cual se caracterizó la mortalidad por enfermedades huérfanas en Chile entre 2002 y 2017, a partir de datos secundarios provenientes de las defunciones individuales registradas en la base oficial del Departamento de Estadística e Información en Salud (DEIS), Ministerio de Salud de Chile, como única fuente secundaria de información.

Para determinar la causa de defunción, se consideró el consenso oficial elaborado en un país de la región con base en la Clasificación Internacional de Enfermedades en su décima revisión (CIE-10) y la homologación de códigos provenientes de Orphanet ^7^^,^^8^, debido a que Chile hasta el momento no ha definido un listado específico de códigos. Tras el proceso de revisión código a código, se identificaron 389 enfermedades que contaban con CIE-10 y 661 eran códigos inespecíficos. Finalmente, se establecieron 389 que tenían códigos específicos o correspondían con el CIE-10 para hacer la búsqueda en el Departamento de Estadística e Información en Salud.

Se excluyeron del análisis 1.185 (0,08 %) defunciones en las cuales no había forma de calcular la edad o no se determinaban las variables de estudio. El análisis univariado se realizó por año de defunción, sexo, grupos de edad según quinquenios, actividad, nivel educativo, región y zona de residencia, y códigos de causa de defunción. Las variables cualitativas se analizaron en función de las frecuencias absolutas y relativas. El tratamiento de las variables cuantitativas se hizo mediante la prueba no paramétrica de Kolmogórov-Smirnov para identificar la normalidad en la distribución de los datos. En correspondencia con lo anterior, se usó la prueba t de Student o la U de Mann-Withney, dependiendo de la distribución observada de los datos de la variable edad ^9^.

Asimismo, se calcularon las tasas de mortalidad específica según sexo y grupos de edad, y se expresaron en muertes por 100.000 personas al año, empleando como denominador las proyecciones de población estimadas por el DEIS correspondientes a los grupos de edad y años de estudio. Se estimaron tasas anuales ajustadas por grupos de edad y, siguiendo el método directo y utilizando como población de referencia la población mundial según la Organización Munial de la Salud (OMS) ^10^.

En el análisis bivariado se buscaron posibles asociaciones entre las características sociodemográficas y la enfermedad huérfana como causa de defunción, considerando este último resultado como como una variable. Para ello, se hizo la prueba de independencia de ji al cuadrado, y un análisis de regresión logística binario para determinar la probabilidad de muerte por esta causa mediante el cálculo de la razón de momios (*Odds Ratio*, OR). El nivel de significación se estableció en menos del 5 %.

Por último, se construyó un modelo de regresión logística multivariado cuyo objetivo era estimar la OR ajustada. Se eligieron las variables independientes para ingresarlas al modelo según la prueba de Hosmer- Lemeshow para modelos bivariados , es decir, aquellas que presentaron un valor de p menor de 0,25 ^11^. Para la selección de los diferentes modelos, se utilizó el método de selección por pasos hacia adelante *(forward)*. En el caso de las variables con más de dos categorías, se usó el criterio de crear tantas variables ficticias (*dummy*) como el número total de categorías menos una*.* La pertinencia de cada variable en el modelo se evaluó mediante la prueba paramétrica de Wald, definiendo como variables significativas aquellas que arrojaran un valor de p menor de 0,05 ^12^.

El procesamiento de los datos y el análisis de la información se realizaron por medio del paquete estadístico R Studio, versión 4.0.3 (2020-10-10), apoyado en las herramientas de Microsoft Office 2019.

### Consideraciones éticas

En el diseño de este trabajo se contemplaron los aspectos éticos generales establecidos por la OMS para la investigación relacionada con la salud de seres humanos, específicamente, a partir de datos secundarios ^13^. Las bases de datos no contenían información de identificación de los fallecidos; en los procedimientos realizados no se intervinieron personas, ni tampoco se incumplió el derecho a la privacidad y los resultados se presentan de manera global.

## Resultados

**Figura 1:**
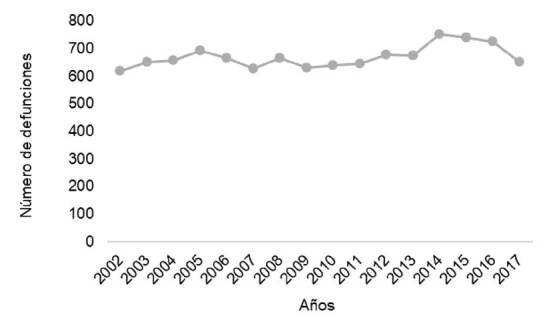
Tendencia de las defunciones por enfermedades en Chile, 2002-2017

En Chile, se registraron alrededor de 1’504.726 defunciones por todas las causas entre los años 2002 y 2017, de las cuales 10.718 se atribuyeron directamente a enfermedades huérfanas, con un promedio de 669,8 muertes anuales. En el 2014, se notificó el mayor número de muertes por esta causa, con un total de 753; mientras que el 2002 fue el año con menor número (618) de decesos. La tendencia global mostró un patrón de ascenso entre el 2003 y el 2016, con una leve reducción en 2017 (figura 1).

Fuente: cálculo a partir de los datos oficiales del Departamento de Estadística e Información en Salud, DEIS, Ministerio de Salud de Chile

Al analizar el comportamiento sociodemográfico de las personas fallecidas debida a este grupo de enfermedades, más de la mitad ocurrió en mujeres (5.604) y el 89,6 % (9.600) se clasificaba en la categoría de inactividad (cuadro 1). En cuanto al nivel educativo, los decesos se presentaron con mayor frecuencia en la población sin ninguna formación académica, con el 49,3 % (5.287).

**Cuadro1.** Características sociodemográficas asociadas a las defunciones debidas a enfermedades huérfanas en Chile, 2002-2017

Variable		Defunciones					
		Enfermedades huérfanas		Otras causas de defunción^†^		X^2^	p
		n	(%)	n	(%)		
Sexo							
	Mujer	5.604	(0,37)	694.312	(46,27)		
	Hombre	5.114	(0,34)	795.656	(53,02)	138,05	0,000
Actividad productiva							
	Inactivo	9.600	(0,64)	1.262.093	(84,10)		
	Activo	1.008	(0,07)	207.228	(13,81)		
	Cesante o desocupado	110	(0,01)	20.647	(1,38)	195,17	0,000
Nivel educativo							
	Superior	490	(0,03)	74.877	(4,99)		
	Medio	1.319	(0,09)	151.462	(10,09)		
	Secundaria	881	(0,06)	283.908	(18,92)		
	Básico o primaria	2.741	(0,18)	798.230	(53,19)		
	Ninguno	5.287	(0,35)	181.491	(12,09)	14.072	0,000
Grupos de edad (años)							
	0-4	4.474	(0,30)	30.017	(2,00)		
	5-9	197	(0,01)	2.945	(0,20)		
	10-14	234	(0,02)	3.812	(0,25)		
	15-19	335	(0,02)	10.324	(0,69)		
	20-24	278	(0,02)	15.208	(1,01)		
	25-29	184	(0,01)	16.448	(1,10)		
	30-34	170	(0,01)	18.914	(1,26)		
	35-39	205	(0.01)	24.147	(1,61)		
	40-44	246	(0,02)	34.188	(2,28)		
	45-49	413	(0,03)	47.185	(3,14)		
	50-54	517	(0,03)	63.378	(4,22)		
	55-59	623	(0,04)	81.920	(5,43)		
	60-64	677	(0,05)	81.542	(6,96)		
	65 y más	2.165	(0,14)	1’037.355	(69,14)	80.889	0,000
Zona de residencia							
	Urbana	9.509	(0,64)	1’286.726	(85,74)		
	Rural	1.209	(0,08)	203.242	(13,54)	50.004	0,000

Fuente: cálculos a partir de los datos oficiales del Departamento de Estadística e Información, DEIS, Ministerio de Salud de Chile

^†^ No se incluyeron 4.040 registros en los que se determinaron las variables de comparación (sexo, actividad productiva, nivel educativo, edad y zona de residencia).

En relación con los grupos de edad más afectados, se encontró una mayor proporción entre los menores de 0 a 4 años, con 41,7 % (4.474), seguido por el de adultos mayores de 65 y más años, con 20,2 % (2.165), y el de 60 a 64 años, con 6,3 % (677). Una gran proporción de ellos residían en la zona urbana, con 88,7 % (9.509).

En cuanto al diagnóstico específico, las causas de muerte no difirieron notoriamente según el sexo. Entre las primeras causas de defunción en las mujeres, sobresalen: enfermedad de Creutzfeldt-Jakob, anencefalia, hepatitis autoinmunitaria, cirrosis biliar primaria, anomalías cromosómicas no especificadas, hernia diafragmática congénita, distrofia muscular, malformaciones congénitas del corazón especificadas, displasia broncopulmonar originada en el periodo perinatal y síndrome de Guillain-Barré (cuadro 2). Mientras que, en la población masculina, se identificaron las siguientes: enfermedad de Creutzfeldt-Jakob, distrofia muscular, anencefalia, hernia diafragmática congénita, anomalías cromosómicas no especificadas, otras malformaciones congénitas del corazón especificadas, displasia broncopulmonar originada en el periodo perinatal, síndrome de Guillain-Barré, trastornos miotónicos y enanismo tanatofórico (cuadro 2). La mediana de la edad fue de 21 años (rango intercuartílico=61).

**Cuadro 2.** Principales causas de mortalidad por enfermedades huérfanas en hombres y mujeres, Chile, 2002-2017

Mujeres			Hombres		
	n	%		n	%
Enfermedad de Creutzfeldt-Jakob	593	10,6	Enfermedad de Creutzfeldt-Jakob	510	10,0
Anencefalia	484	8,6	Anencefalia	426	8,3
Hepatitis autoinmunitaria	381	6,8	Distrofia muscular	436	8,5
Cirrosis biliar primaria	267	4,8	Hernia diafragmática congénita	277	5,4
Anomalía cromosómica, no específica	238	4,2	Anomalía cromosómica, no específica	241	4,7
Hernia diafragmática congénita	230	4,1	Otras malformaciones congénitas del corazón, especificadas	179	3,5
Distrofia muscular	135	2,4	Displasia broncopulmonar originada en el periodo neonatal	178	3,5
Otras malformaciones congénitas del corazón, especificadas	130	2,3	Síndrome de Guillain-Barré	171	3,3
Displasia broncopulmonar originada en el periodo neonatal	116	2,1	Hepatitis autoinmunitaria	71	1,4
Síndrome de Guillain-Barré	110	2,0	Miastenia gravis	63	1,2
Miastenia gravis	107	1,9	Cirrosis biliar primaria	20	0,4
Otras enfermedades huérfanas	2.813	50,2	Otras enfermedades huérfanas	2.542	49,7
Total	5.604	100	Total	5.114	100

Fuente: cálculo a partir de los datos oficiales del Departamento de Estadística e Información en Salud, DEIS, Ministerio de Salud de Chile

Dado que los datos correspondientes a la variable edad no provenían de una distribución normal tanto en hombres como en mujeres (p<2.2e-16, prueba Kolmogórov-Smirnov), se encontró que existían diferencias estadísticas (p<2.2e-16, prueba U de Mann-Whitney) en la mediana de la edad en ambos sexos, esa diferencia es positiva a favor de los hombres.

**Figura 2:**
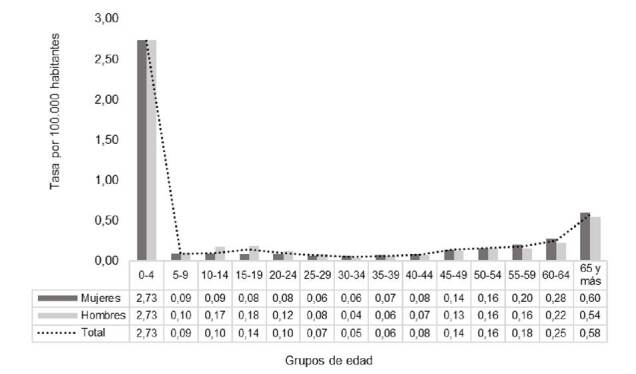
Tasa de mortalidad media anual debida a enfermedades huérfanas según sexo y grupos de edad, Chile, 2002-2017

La tasa media de mortalidad debida a enfermedades huérfanas en Chile para el periodo fue de 3,9 por 100.000 habitantes: 4,1 en mujeres y 3,8 en hombres. Las tasas más altas se observaron en los menores de 0 a 4 años en ambos casos. En general, las mujeres registraron las mayores tasas, principalmente a partir de los 35 años de edad. En hombres, las tasas fueron más altas a partir del grupo de 5 a 9 años y hasta el de 25 a 29 años respecto de las mujeres (figura 2). Las tasas que predominaron en hombres fueron las que se presentaron a partir del grupo de 45 a 49 años.

Fuente: cálculo a partir de los datos oficiales del Departamento de Estadística e Información en Salud, DEIS, Ministerio de Salud de Chile

Con respecto la región de residencia, los resultados indicaron que las regiones Metropolitana de Santiago (4.174), Valparaíso (1.159), Biobío (1.035), Maule (607) y Araucanía (623), concentraron el 70,8 % de los fallecimientos atribuidos a estas causas.

Al ajustar las tasas por sexo y edad, la situación por región varía a través de los años. La región de Tarapacá aportó la tasa media ajustada anual más alta, con 6,5 por 100.000 habitantes por año, observándose un incremento del 2006 al 2013 y del 2015 al 2017 con tasas superiores al promedio nacional, seguido por Antofagasta con 3,3. Las demás regiones, arrojaron tasas iguales y menores de 0,6 muertes por 100.000 habitantes (cuadro 3).

**Cuadro 3.** Tasa de mortalidad debida a enfermedades huérfanas, ajustadas por edad, según región de residencia, Chile, 2002-2017

Regiones	Tasa ajustada de mortalidad por 100.000 personas/año																
	2002	2003	2004	2005	2006	2007	2008	2009	2010	2011	2012	2013	2014	2015	2016	2017	Media
Chile	3,9	4,1	4,1	4,3	4,1	3,8	4,0	3,7	3,8	3,7	3,9	3,8	4,2	4,1	4,0	3,5	3,9
Tarapacá	4,8	5,0	4,0	2,0	5,0	8,0	8,0	8,0	4,0	9,0	10,0	10,0	4,0	10,0	7,0	6,0	6,5
Antofagasta	6,0	3,1	1,5	1,5	3,0	2,9	6,7	5,5	2,7	3,4	6,7	4,1	2,4	1,6	3,3	3,3	3,6
Atacama	4,0	0,0	0,0	0,0	0,0	0,0	0,0	0,0	0,0	0,0	0,0	0,0	0,0	0,0	0,0	0,7	0,3
Coquimbo	4,0	0,0	0,0	0,0	0,4	0,0	0,0	0,7	0,0	0,0	0,0	0,0	0,0	0,0	0,0	0,0	0,3
Valparaiso	2,0	0,0	0,0	0,0	0,0	0,0	0,0	0,0	0,0	0,0	0,0	0,7	0,7	0,7	0,7	0,7	0,3
Libertador B. O'Higgins	2,0	0,0	0,0	0,4	0,0	0,7	0,0	0,0	0,0	0,0	0,0	0,0	0,0	0,6	0,0	0,0	0,2
Maule	3,0	0,0	0,0	0,0	0,0	0,0	0,0	0,0	0,0	0,0	0,0	0,0	0,0	1,1	1,1	0,5	0,4
Biobío	3,0	0,0	0,0	0,0	0,0	0,0	0,0	0,0	0,0	0,0	0,0	0,0	0,0	0,0	0,0	0,0	0,2
Araucanía	3,0	0,0	0,0	0,0	0,0	0,0	0,0	0,5	0,0	0,0	0,5	0,0	0,0	0,5	0,0	0,0	0,3
Los Lagos	3,0	0,0	0,0	0,0	0,0	0,0	0,0	0,0	0,0	0,6	0,6	1,1	0,5	1,5	0,0	0,5	0,5
Aisén del Gral. C. Ibáñez del Campo	6,0	0,0	0,0	0,0	0,0	0,6	0,0	0,0	0,0	0,0	0,6	0,0	0,0	0,0	0,0	0,0	0,5
Magallanes y de la Antártida Chilena	8,0	0,0	0,0	0,0	0,3	0,0	0,6	0,0	0,0	0,0	0,0	0,0	0,0	0,0	0,0	0,0	0,6
Metropolitana de Santiago	3,0	0,0	0,0	0,0	0,0	0,7	0,0	0,0	0,0	1,1	0,0	0,5	0,0	0,5	0,0	0,5	0,4
Los Ríos	5,0	1,9	0,0	0,0	0,5	0,0	0,0	0,0	0,8	0,0	0,0	0,7	0,0	0,0	0,0	0,0	0,6
Arica y Paniracota	4,0	0,0	0,6	0,0	0,5	0,0	0,0	0,0	0,0	1,8	0,8	0,0	0,0	0,7	0,7	0,0	0,6
Nuble	3,0	0,0	0,9	0,5	0,0	1,0	0,0	0,0	0,0	0,9	0,0	0,8	0,0	0,7	0,0	0,0	0,5

Fuente: cálculos a partir de los datos oficiales del Departamento de Estadística e Información, DEIS, Ministerio de Salud de Chile

En el análisis bivariado entre variables cualitativas, se encontró que estuvieron asociados a los fallecimientos por enfermedad huérfana el sexo, la actividad, el nivel educativo, la edad distribuida por grupos y la zona de residencia (cuadro 1). Por su parte, la regresión logística bivariada (OR cruda) arrojó un valor estadísticamente significativo para algunas de las características previamente estudiadas, las cuales fueron corroboradas mediante el análisis de regresión logística multivariado (OR ajustado).

Al considerar el sexo, se determinó que las mujeres tienen 1,75 más posibilidades de fallecer por enfermedades huérfanas, en comparación con los hombres (OR ajustado=1,75; IC_95%_ 1,69-1,82). Las personas en condiciones de inactividad mostraron una probabilidad de morir de 1,32 veces más (_a_OR = 1,32; IC_95%_ 1,09-1,59). Con respecto al nivel educativo, se encontró que los individuos sin ningún nivel de formación académica tenían hasta 4,31 veces más probabilidades de fallecer a causa de alguna de estas enfermedades (_a_OR = 4,31; IC_95%_ 3,93-4,73).

En cuanto a los grupos de edad, se corroboró que la población pediátrica (0 a 4 años) sigue presentando el mayor riesgo de morir (_a_OR = 15,30; IC_95%_ 14,10-19,20), en comparación con los de 5 a 9 (_a_OR = 6,91; IC_95%_ 6,00-9,05) y de 10 a 14 años (_a_OR = 6,37; IC_95%_ 5,51-8,00). De igual manera ocurre con las personas de 15 a 19 y 20 a 24 años, respectivamente (_a_OR = 3,57; IC_95%_ 3,40-4,35 *Vs*. _a_OR = 2,6; IC_95%_ 1,68-2,48). Además, se observó que los fallecidos que residían en el casco urbano tenían 1,22 veces más probabilidades de morir por estas causas, frente a los que residen en zonas rurales (_a_OR = 1,22; IC_95%_ 1,10-1,29) (cuadro 4).

**Cuadro 4:** Análisis de regresión logística bivariado y multivariado del riesgo de mortalidad debida a enfermedades huérfanas en Chile, 2002-2017

Variables		Análisis bivariado			Análisis multivariado		
		OR cruda	IC95%	p	OR ajustado	IC95%	p
Sexo							
	Mujer	1,26			1,75		
	Hombre*	1	1,21-1,3	<0,001	1	1,69-182	<0,001
Actividad							
	Cesante o desocupado	1			1		
	Inactivo	1,43	1,18-1,72	<0,001	1,32	1,09-1,59	0,004
	Activo	0,91	0,71-1,11	0,366	0,9	0,74-1,09	0,287
Nivel educativo		1			1		
	Superior*	1			1		
	Medio	1,33	1,2-1,48	<0,001	1,33	1,2-1,48	<0,001
	Secundaria	0,47	0,42-0,53	<0,001	0,46	0,41-0,52	<0,001
	Básico o primaria	0,52	0,48-0,58	<0,001	0,51	0,46-0,56	<0,001
	Ninguno	4,45	4,06-4,89	<0,001	4,31	3,93-4,73	<0,001
Grupos de edad (años)							
	0-4	16,58	14,21-19,35	<0,001	15,30	14,10-19,20	<0,001
	5-9	7,44	6,04-9,17	<0,001	6,91	6,00-9,05	<0,001
	10-14	6,83	5,59-8,35	<0,001	6,37	5,51-8,00	<0,001
	15-19	3,61	3,4-4,35	<0,001	3,57	3,4-4,35	<0,001
	20-24	2,03	1,68-2,46	<0,001	2,06	1,68-2,48	<0,001
	25-29	1,24	1,01-1,53	0,041	1,26	1,05-1,55	0,029
	30-34*	1					
	35-39	0,94	0,77-1,16	0,584	0,93	0,70-1,10	0,488
	40-44	0,8	0,66-0,97	0,026	0,8	0,66-0,97	0,011
	45-49	0,97	0,81-1,17	0,772	0,93	0,79-1,15	0,452
	50-54	0,91	0,76-1,08	0,275	0,86	0,71-1,07	0,101
	55-59	0,85	0,72-1,01	0,062	0,81	0,70-1,01	0,013
	60-64	0,72	0,61-0,85	<0,001	0,68	0,60-0,81	<0,001
	65 y más	0,23	0,2-0,27	<0,001	0,21	0,19-0,27	<0,001
Área de residencia							
	Rural*	1					
	Urbana	1,24	1,17-1,32	<0,001	1,22	1,10-1,29	<0,001

Fuente: cálculos a partir de los datos oficiales del Departamento de Estadística e Información, DEIS, Ministerio de Salud de Chile

OR: razón cruda de momios; OR ajustado: razón ajustada de momios

^*^ Categoría de referencia

## Discusión

Entre el 2002 y el 2017, se registraron 10.718 defunciones directamente relacionadas con enfermedades huérfanas en Chile. No obstante, estas cifras podrían ser significativamente mayores de lo que muestran los hallazgos presentados en este trabajo, dadas las limitaciones atribuidas a su clasificación en todo el mundo. Esto significa que muchas de ellas puedan ser ignoradas o clasificadas de manera inapropiada en la causa de defunción a partir de las complicaciones derivadas ^14^^,^^15^.

El análisis de las características sociodemográficas revela varios elementos importantes que ayudan a entender la amplia diversidad de los trastornos raros ya que su comportamiento varía, no solo de enfermedad a enfermedad, sino también, dentro del mismo contexto al que pertenecen las personas afectadas. Según los hallazgos, una proporción representativa (52,3 %) de estas defunciones se presentaron en mujeres. Estos resultados coinciden con los datos oficiales documentados por el Sistema Nacional de Vigilancia en Salud Pública en Colombia cuya tendencia ha mostrado un crecimiento más acentuado en este grupo de población en los últimos cinco años ^16^.

En parte, esto podría explicarse porque un porcentaje importante de estas afecciones son de origen autoinmunitario y se ha demostrado que las alteraciones del sistema inmunológico predominan en las mujeres ^17^. Sin embargo, en otro estudio en que se analizaron las muertes por estas causas en ese mismo país, se reveló que el 51,4 % (3.468) de ellas ocurrieron en hombres de todas las edades ^18^. El carácter incapacitante de las enfermedades huérfanas guarda relación, no solamente con su naturaleza crónica, sino también, con el alto grado de discapacidad que generan y, a su vez, con una importante vulnerabilidad económica que coloca a los pacientes y sus familias ante un amplio panorama de necesidades.

Esto se refleja de alguna manera en el elevado porcentaje (89,4 %) de fallecidos que se encontraban en condiciones de inactividad, sobre todo en menores de cinco años y en adultos mayores de 65 años, a quienes se les atribuye el 61,9 % (6.639) de las muertes identificadas. Esta tendencia es coherente con lo informado en la literatura científica sobre morbilidad y mortalidad prematura ^19^^-^^21^. Respecto al nivel educativo, la mayor proporción de las muertes se concentró en poblaciones sin ninguna escolaridad.

El comportamiento de las enfermedades huérfanas es muy heterogéneo. En el caso de los niños, representa una reducción de sus capacidades físicas, habilidades mentales, y destrezas sensoriales y de comportamiento. La falta de escolarización se podría entender como un factor de riesgo potencial para morbimortalidad ^22^. Las malformaciones congénitas constituyen las primeras causas de muerte en Chile. Este patrón coincide con la situación reportada en la mayoría de los países de Latinoamérica, cuyas muertes en menores de un año tienden a incrementar año tras año, ocupando entre el segundo y el quinto lugar de los fallecimientos; esto equivale al 27 % de la mortalidad en menores de cinco años ^23^. En otro estudio colombiano, se revelaron cifras cercanas a 22.361 fallecimientos perinatales atribuibles malformaciones congénitas, con tasas que oscilaron entre 205,81 y 74,18 por 10.000 nacidos vivos durante 10 años estudiados ^24^.

En relación con los diagnósticos específicos, la anencefalia constituye la primera causa de muerte en los menores de 0 a 4 años de ambos sexos; este dato concuerda con lo documentado en Irlanda en neonatos de 0 a 28 días, cuyas defunciones aportaron el 51,58 % de la carga de mortalidad infantil de ese país entre 2006 y 2016 ^25^.

La enfermedad de Creutzfeldt-Jakob se ubicó entre las enfermedades huérfanas de mayor frecuencia en la población de 15 años y en los mayores de 65 años, principalmente mujeres. En los adultos de 65 y más años, la enfermedad puede llegar a volverse más prevalente como causa principal de demencia, incrementándose su gravedad con la edad. En un estudio reciente en Japón, se reveló un incremento significativo en la mortalidad (3,2%; IC_95%_ 1,4-5,1) y la incidencia (6,4 %; IC_95%_ 4,7-8,1), especialmente entre los adultos mayores de 70 años ^26^.

La distrofia muscular fue una de las enfermedades que más afectó a los hombres de todas las edades en Chile. Existe evidencia, especialmente, de la variante hereditaria de Duchenne, un tipo de enfermedad neuromuscular progresiva que afecta uno de cada 5.000 niños varones nacidos vivos _(_^27^_)_. Otra enfermedad común en ambos sexos fue la de Guillain-Barré. No fue más frecuente en la población masculina, comportamiento que coincide con lo reportado en otra investigación colombiana _(_^28^_)_.

Los hallazgos en cuanto a edad también difieren de los documentados en Perú, en donde la mediana de la edad fue de 47 años, situación que demuestra que las enfermedades huérfanas constituyen un grupo muy variado y complejo que afecta de forma distinta a las personas que la padecen ^29^. Se observaron diferencias en las tasas globales de mortalidad según el sexo y los grupos de edad, cuyos resultados concuerdan parcialmente con los reportados en la Comunidad de Madrid, España, entre 1999 y 2003, donde estas enfermedades cobraron más vidas en varones de todos los grupos de edad, mientras que el máximo riesgo de muerte se identificó en los menores de un año ^30^. La situación de la mortalidad por este grupo de enfermedades reveló, en esa misma comunidad española, una tendencia al incremento en los años 2010 y 2012, hasta alcanzar una tasa media anual de 15,4 por 100.000, con mayor afectación en las mujeres mayores de 75 años, con 170,8 por 100.000, en comparación con los hombres de ese mismo grupo, con 70,4 por 100.000 ^31^. Las mayores tasas en Chile se registraron en las mujeres, particularmente en los extremos de la vida. El riesgo de morir por alguna enfermedad huérfana fue mayor en los primeros años de vida de ambos sexos.

El comportamiento de las defunciones mostró diferencias regionales importantes según la región de residencia de los fallecidos: las regiones Metropolitana de Santiago, Valparaíso, Biobío, Maule y Araucanía, concentraron la mayor proporción de las muertes. En parte, este patrón podría explicarse por la limitada disponibilidad de talento humano calificado en salud, la distribución de los laboratorios de diagnóstico y la de los servicios de salud de alta complejidad en el manejo de estas enfermedades en todo el país.

Según el Ministerio de Salud, 47 médicos (50 % genetistas clínicos y 40 % neurólogos infantiles) se dedicaban al diagnóstico y al tratamiento de personas con enfermedades huérfanas en Chile en el 2019. El 80 % de los genetistas y la mayoría de los hospitales públicos, centros privados, universidades o institutos especializados, están ubicados en la Región Metropolitana de Valparaíso y Biobío. En contraste, 10 regiones no disponen de esta especialidad médica ^32^^,^^33^. Las mayores tasas de mortalidad se registraron en Tarapacá y Antofagasta, lo cual es coherente con el efecto de las diferencias en la estructura de la edad por región que influyen en el cálculo del indicador sobre la tendencia nacional y regional.

Se encontró una asociación estadísticamente significativa entre las defunciones por enfermedades huérfanas con el sexo, la actividad, el nivel educativo, el grupo de edad y la zona de residencia, resultados que fueron corroborados mediante el análisis de regresión logística multivariada. El análisis de los modelos lineales generalizados mostró que el riesgo de morir por enfermedades huérfanas en las mujeres del grupo de 65 o más años que residían en zonas urbanas, fue 1,75 veces mayor, en comparación con los hombres. Este hallazgo coincide con el reportado en un estudio de base hospitalaria en el norte del Perú (p<0,001; RPa (razón de prevalencia ajustada): 1,76; IC_95%_ 1,67-1,86) ^29^. Mientras que otro análisis en la región de Toscana, en el norte de Italia, hecho entre 2010 y 2018, reveló un incremento significativo en el riesgo de muerte de la población masculina, 1,48 veces mayor que el de las mujeres (*Hazard Ratio*, HR=1,48; IC_95%_ 1,38-1,58) _(_^34^_)_.

Se observó un mayor riesgo de muerte en los grupos inactivos, lo cual podría responder entre otras razones, a la naturaleza crónicamente debilitante de estas enfermedades, la cual acarrea discapacidades múltiples y graves. Este hallazgo guarda estrecha relación con el riesgo de morir entre las personas sin ningún grado de escolaridad; en ellas, se incrementa hasta cuatro veces más que en aquellos que tenían algún nivel de escolaridad. Estos resultados difieren notoriamente con lo reportado en Cali (Colombia), cuyos análisis demuestran que el nivel de educativo no presentaba ninguna relación estadísticamente significativa con la mortalidad por estas causas ^28^.

Por otra parte, el riesgo de morir aumenta hasta 15 veces en los infantes en edades entre 0 y 4 años con respecto al resto de la población; esto se atribuye, principalmente, a alteraciones estructurales y fisiológicas muy amplias y complejas asociadas con anomalías genéticas originadas durante las etapas tempranas del desarrollo embrionario ^35^^,^^36^. En el mismo trabajo realizado en Cali (Colombia), el mayor riesgo de mortalidad se presentó en los individuos de 30 a 44 años (p<0,03; RPa: 14,07; IC_95%_ 1,23-160,4) ^28^. Estas diferencias reflejan la amplia variabilidad en el perfil de morbilidad y mortalidad de las enfermedades huérfanas entre países, incluso dentro de una misma región. El riesgo de muerte fue significativamente mayor (_a_OR = 1,22; IC_95%_ 1,10-1,29) en las personas que habitualmente residían en zonas urbanas, en comparación con aquellas que vivían en áreas más dispersas o rurales. Esto corrobora lo que se conoce sobre las posibilidades de acceso al diagnóstico oportuno y el manejo adecuado en las grandes ciudades capitales del país ^32^.

Como limitaciones de esta investigación, cabe mencionar que tanto los cálculos del indicador de mortalidad como la medición del riesgo de fallecer por este grupo de enfermedades, se hicieron a partir de información secundaria proveniente de las bases de defunciones oficiales y no se tuvieron en cuenta otras fuentes que ayudaran a contrastar la información entre distintas bases de datos y de esta manera identificar posibles muertes que no se encontraran reportadas en el sistema nacional de estadísticas vitales chileno. El eventual subregistro y la falta de especificidad o uniformidad de los diagnósticos pueden afectar el cálculo final de las tasas de mortalidad, con lo cual podría estar subestimada la magnitud real de los fallecimientos por enfermedades en Chile.

Otra limitación para considerar es que, en el análisis multivariado, no se empleó la estrategia metodológica de estratificación para la variable actividad, lo que podría haber sobreestimado el riesgo de morir para esta variable dado el alcance de los datos. De igual manera, no se hizo este procedimiento con las defunciones en menores de 0 a 4 años de edad. Esto significa que el riesgo observado en este grupo de población específica también podría estar arrojando valores más altos, ya que la mortalidad perinatal y la neonatal tardía varían notoriamente entre ellos.

Aun con todas estas limitaciones, los resultados presentados en este trabajo muestran un panorama actualizado sobre la situación de la mortalidad por enfermedades huérfanas en su conjunto y las principales causas de muerte en la población atribuidas a diagnósticos específicos relacionados con este grupo de enfermedades. Por lo tanto, se constituyen en un insumo para seguir profundizando en el tema y priorizar intervenciones en aquellas regiones y poblaciones más afectadas, ya que la mayoría de ellas son muertes prematuras o antes de cumplir la esperanza de vida. Además, se podría decir que este análisis es uno de los primeros en este campo que se centra en las muertes debidas a este grupo de enfermedades de forma global y explora los posibles factores asociados a la mortalidad en nuestro medio.

Se concluye que la mortalidad por enfermedades huérfanas, en su conjunto, se perfila como problema creciente de salud pública en Chile.

Los hallazgos demuestran la importancia de realizar más estudios, pero, sobre todo, de avanzar en la estandarización de un sistema de codificación y clasificación propio, de acuerdo con los perfiles de morbilidad y mortalidad asociados con enfermedades huérfanas en nuestro contexto, que proporcione información oportuna y de calidad para la toma de decisiones y oriente las intervenciones en salud pública.
